# Faecal microbiota transplant in Parkinson’s disease: pilot study to establish safety & tolerability

**DOI:** 10.1038/s41531-025-01061-5

**Published:** 2025-07-09

**Authors:** Michele De Sciscio, Robert V. Bryant, Sarah Haylock-Jacobs, Alice S. Day, William Pitchers, Robert Iansek, Samuel P. Costello, Thomas E. Kimber

**Affiliations:** 1 Neurology Unit, Royal Adelaide Hospital, Central Adelaide Local Health Network, Adelaide, Australia; 2 Gastroenterology Unit, Queen Elizabeth Hospital, Central Adelaide Local Health Network, Adelaide, Australia; 3 Faculty of Health & Biomedical Sciences, University of Adelaide, Adelaide, Australia; 4 Basil Hetzel Institute, Adelaide, Australia; 5 BiomeBank, Adelaide, Australia; 6 Clinical Research Centre for Movement Disorders and Gait, Kingston Centre, Parkinson’s Foundation Centre of Excellence, Monash Health, Victoria, Australia

**Keywords:** Parkinson's disease, Basal ganglia

## Abstract

Emerging evidence suggests gut microbiota differences in Parkinson’s Disease (PD) may impact disease progression and treatment. Faecal Microbiota Transplantation (FMT) offers a potential therapeutic approach. We conducted an open-label pilot study to assess the safety, tolerability, and symptom impact of FMT in 12 patients with mild to moderate PD, administered via enema for 6 months. FMT was safe and well tolerated, causing only mild, transient gastrointestinal symptoms. While no significant motor symptom changes were observed, there was a trend toward reduced daily OFF time at 2 months. Whilst no sustained improvement in non-motor symptoms was found after 6 months, transient improvements in quality of life and non-motor scores were noted at 2 months; these gains regressed by study end. Overall, extended FMT therapy in PD appears safe and tolerable, with reduction in daily motor OFF time and self-reported non-motor symptoms that was not sustained throughout the 6-months of treatment

## Introduction

Parkinson’s Disease (PD) is characterized by the accumulation of abnormal alpha-synuclein isoforms in neurons, causing dysfunction and cell death^1^. Disease progression is thought to result from the cell-to-cell transmission of alpha-synucleinopathy, leading to neurodegeneration^2^. The hallmark motor symptoms—tremor, rigidity, and bradykinesia—reflect neurodegeneration in central nervous system (CNS) motor circuits. However, evidence suggests that alpha-synuclein pathology begins in non-CNS neurons, such as those in the gut, years before affecting CNS neurons^3^.

The gut is increasingly recognized as a key area in PD research. Alpha-synucleinopathy affects enteric neurons early, often causing constipation as a prodromal symptom^4^. Additionally, the vagus nerve may facilitate the spread of alpha-synucleinopathy from the gut to the CNS, with reduced PD risk in individuals who have undergone vagotomy and increased risk in those with inflammatory bowel disease^5^. This has led to growing interest in the role of gut microbiota in PD pathogenesis. Studies show that PD patients have altered microbiota, including higher levels of pro-inflammatory bacteria, lower levels of anti-inflammatory bacteria, and reduced microbial diversity^6–8^. Though the exact link between gut dysbiosis and PD remains unclear, it may involve increased gut permeability and alpha-synuclein seeding in enteric neurons^2^. Moreover, microbiota composition correlates with motor and non-motor symptom severity and responses to dopaminergic treatments^9^.

Faecal Microbiota Transplantation (FMT) is emerging as a potential therapy to modulate the gut microbiota in PD. Parkinson’s Disease mouse models have shown that FMT with healthy human-derived microbes improves motor function and protects against dopaminergic neuronal death^10^. However, human studies on FMT’s safety and efficacy are limited to short-term trials^11,12^.

This study aims to assess the safety and tolerability of an extended 6-month FMT course in mild to moderate PD patients (primary outcomes). We will also evaluate the impact of FMT on daily OFF time, motor and non-motor symptoms, quality of life, and changes in gut microbiota composition and function (secondary outcomes).

## Results

### Patient Characteristics

A total of 12 subjects (8 females, 4 males; mean age 69.5 years) received FMT treatment, Table 1. The mean disease duration was 6.9 years, and all were on L-dopa therapy ± adjunctive dopaminergic treatments, with a mean daily L-dopa equivalent dose of 684 mg (range 100–1300 mg). Key clinical markers of PD severity included: (i) daily OFF time (mean 4.8 h/24 h, SD 2.2), (ii) UPDRS Part 3 in the OFF state (mean 28.3, SD 12.5) and ON state (mean 12.7, SD 8.3), and (iii) total UPDRS score (mean 42.8, SD 15.5). Constipation was reported in 58% of patients, with 9 of 12 subjects (75%) meeting the Rome IV criteria for functional constipation based on baseline 7-day bowel diaries.

**Table 1:** Patient Demographics and Baseline Values

Baseline Characteristics	*n* = 12
Male Patients	8 (66%)
Age at enrolment (years)	69.5 (4.3)
Age at diagnosis (years)	62.6 (5.7)
BMI	25.0 (5.6)
Disease duration at enrolment, (years)	6.9 (2.4)
Medications	
**­** - L-dopa equivalent daily dose (mg) - Dopamine agonist ** - ­**Monoamine oxidase inhibitors - COMT inhibitors	684 (431) 8 (67%) 7 (58%) 4 (33%)
Comorbidities	
­ - Diabetes ­ - Hypertension ­ - Depression ­ - Anxiety	2 (17%) 3 (25%) 4 (33%) 5 (42%)
Parkinson’s symptoms	
­ - Tremor ­ - Rigidity ­ - Akinesia/Bradykinesia ­ - Freezing of gait ­ - Constipation	10 (83%) 11 (92%) 12 (100%) 3 (25%) 7 (58%)
Montreal Cognitive Assessment	27.3 (1.3)
Parkinson’s Disease Severity Metrics	
­ - UPDRS 1 ­ - UPDRS 2 ­ - UPDRS 4 ­ - UPDRS 3: OFF-state ­ - UPDRS 3: ON-state ­ - Hauser Motor Diary (OFF time/24 h)	2.3 (1.6) 21.4 (8.0) 6.3 (1.8) 28.3 (12.5) 12.7 (8.3) 4.8 (2.2)

Data expressed as either mean (SD), or number of patients (%). *BMI* Body Mass Index, *COMT* catechol-O-methyltransferase, UPDRS Unified Parkinson’s Disease Rating Scale.

* Constipation determined according to the Rome IV Criteria for Colonic Disorders

### Safety and Tolerability

All subjects who received at least one dose of FMT were included in the safety analysis. Eleven of 12 subjects (92%) completed the full 6-month treatment course. One serious adverse event (SAE) occurred: a subject was hospitalized with worsening motor symptoms after 2 months of treatment and withdrew from the study. The SAE was deemed unlikely to be related to FMT, as the subject had a history of steady functional decline prior to enrolment.

Gastrointestinal adverse events were reported by 10 of 12 subjects (83%) as ‘probably’ or ‘definitely’ related to FMT, Table 2. These events were transient and mild, mostly occurring during the induction phase. No treatments were withheld due to adverse events. The most common adverse events were increased flatulence (50%), abdominal pain (42%), constipation (42%), and bloating (42%).

**Table 2:** Self-reported adverse events throughout 6 months of therapy deemed “probably” or “definitely” related to FMT

Adverse event	Number of Patients (%)
Nausea	3 (25%)
Bloating	5 (42%)
Abdominal pain/cramping	4 (33%)
Diarrhoea	3 (25%)
Constipation	7 (58%)
Increased flatulence	6 (50%)

**Fig. 1:**
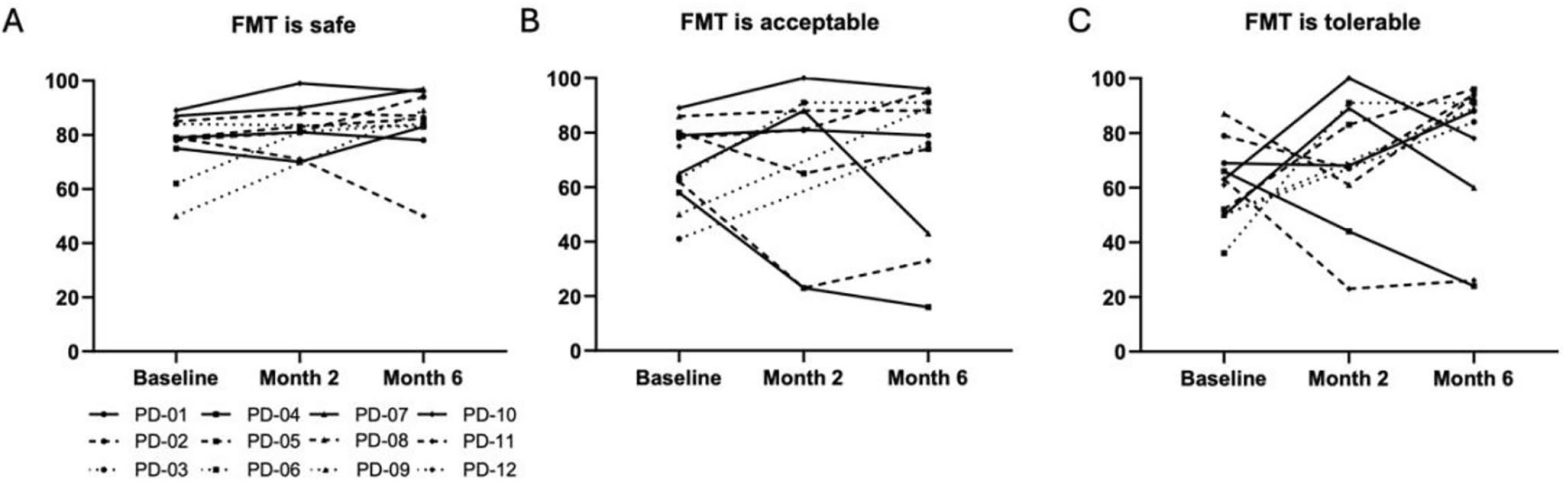
Safety, acceptability, and tolerability of FMT. Self-reported safety (**A**), acceptability (**B**), and tolerability (**C**) of FMT in 12 patients with mild to moderate PD across 3 timepoints (baseline, 2-months, and 6-months) assessed using a 100-point visual analogue scale. The majority of patients rated FMT as highly acceptable and safe, with 11 out of 12 participants scoring above 70/100 at both the 2- and 6-month timepoints.

Using a 100-point visual analogue scale, most patients rated the acceptability and safety of FMT highly, with scores above 70/100 in 11 of 12 patients at both 2 and 6 months, Fig. 1.

### Efficacy of FMT on Motor Function

**Fig. 2:**
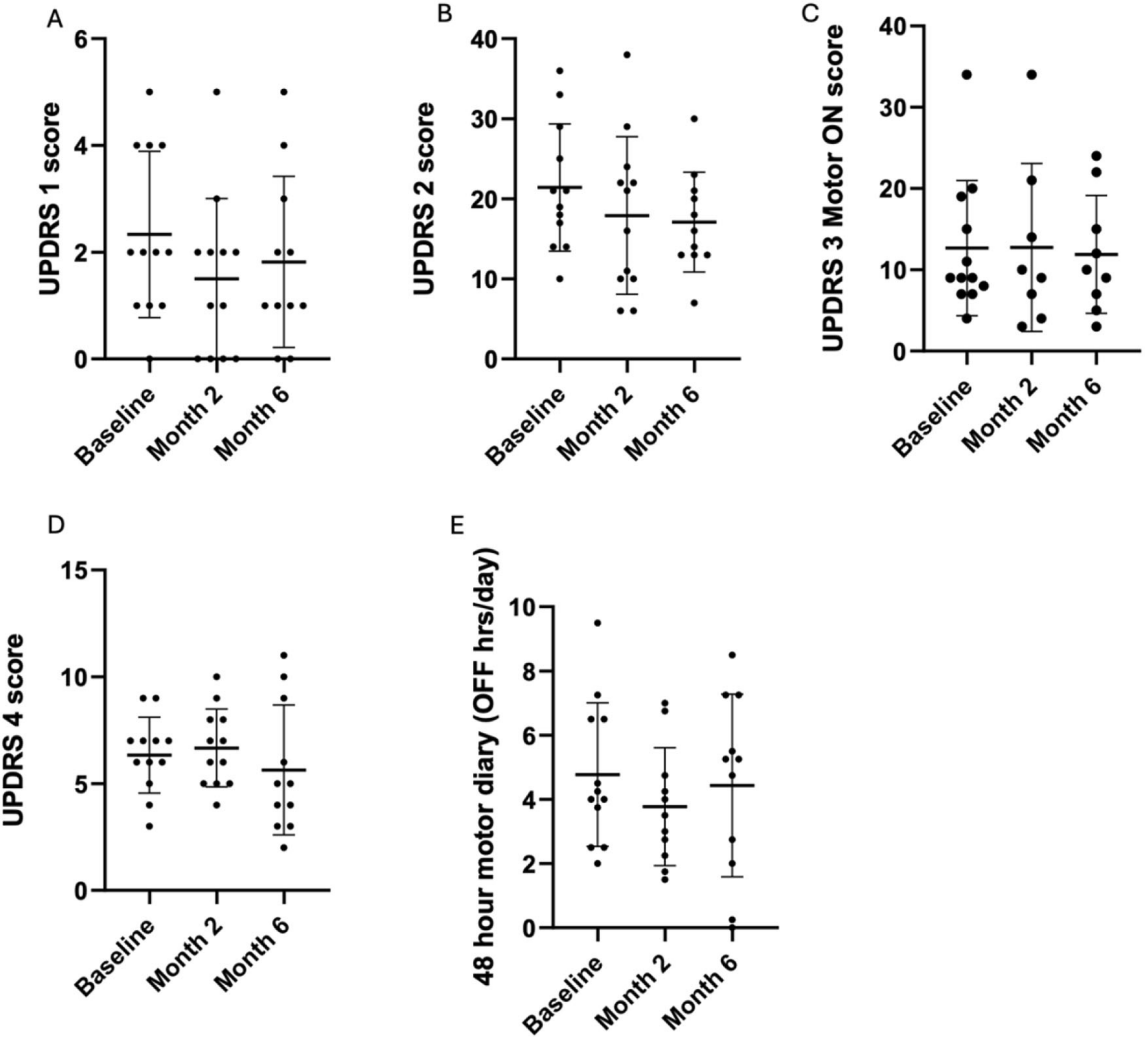
Motor and non-motor assessments at 2 and 6-months following FMT in patients with mild to moderate PD. At 2 months, there was a non-statistically significant reduction in the mean UPDRS Part I score (**A**) from a baseline of 2.3 (SD 1.6) to 1.5 (SD 1.5), representing a 35% reduction (p = 0.336). Similarly, the UPDRS Part II score (**B**) decreased from 21.4 (SD 8.0) to 17.9 (SD 9.8), a 16% reduction (p = 0.089). While the reduction in Part II scores was maintained through the end of the study, Part I scores regressed toward baseline levels. No significant changes were observed in UPDRS Part III (**C**) or Part IV (**D**) scores across the three time points. Patients reported a reduction in daily OFF time at 2 months (3.8 h/day from a baseline of 4.8 h/day), which did not reach statistical significance (p = 0.58), and this effect was not sustained by the end of the study (4.4 h/day, **E**).

The mean self-reported daily OFF-time (averaged over 48 h) was 4.8 h/day (SD 2.2) at baseline and reduced to 3.8 h/day (SD 1.8) after 2 months (*p* = 0.58). However, this reduction was not sustained, with daily OFF-time returning to baseline levels by 6 months (4.4 h/day, SD 2.8), Fig. 2. There was no significant change in UPDRS-Part IV scores, assessing motor complications, from baseline (6.3, SD 1.8) to 2 months (6.7, SD 1.8) and 6 months (5.6, SD 3.0) (*p* = 0.49).

Most subjects (7/12) attended their 2- and 6-month reviews in a motor ON-state, allowing comparisons of ON-state MDS-UPDRS III (motor examination) scores across all three timepoints. While there was no improvement in these scores (baseline 9.4, SD 5.2; 2 months 9.7, SD 6.2; 6 months 9.7, SD 6.2; p = 0.873), there was also no deterioration in the mean MDS-UPDRS III score over the 6-month treatment period, Fig. 2.

### Efficacy of FMT on Non-Motor Function

After two months of FMT therapy, there was a non-significant reduction in the mean UPDRS Part I score (baseline 2.3, SD 1.6 vs 2 months 1.5, SD 1.5; *p* = 0.336, 35% reduction) and UPDRS Part II score (baseline 21.4, SD 8.0 vs 2 months 17.9, SD 9.8; *p* = 0.089, 16% reduction), Fig. 2. This reduction was maintained at 6 months for UPDRS Part II (17.1, SD 6.2), but UPDRS Part I showed regression towards baseline (1.8, SD 1.6).

**Fig. 3:**
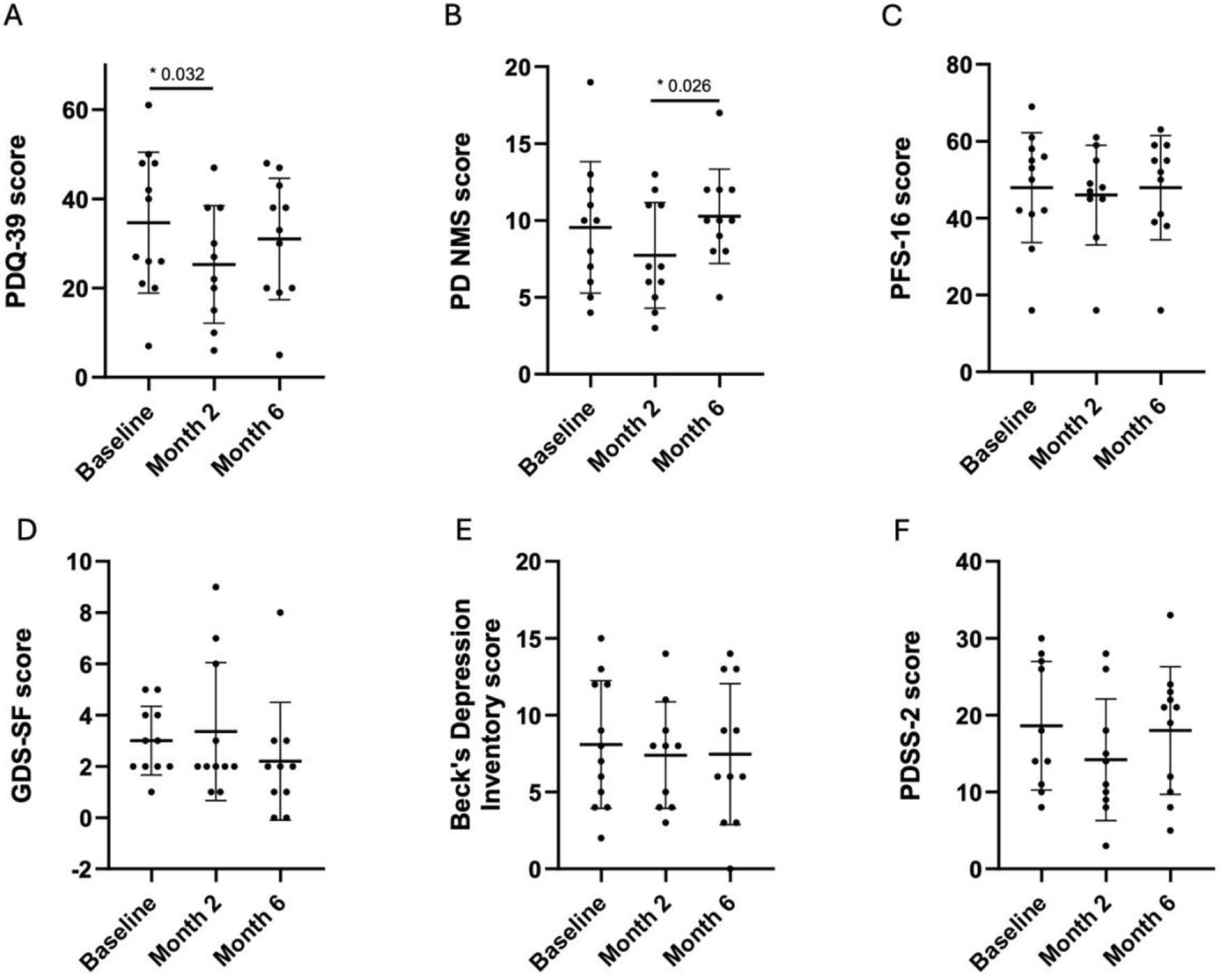
Comparison of Non-Motor Questionnaires at 2- and 6-Months Post-FMT with Baseline. Patients reported an improvement in several non-motor symptoms of PD following FMT, as evidenced by a significant reduction in the PDQ-39 score at both 2 and 6 months compared to baseline, with statistical significance reached (p = 0.032) (Panel **A**). A trend toward improvement was observed in the NMSQ score at 2 months (p = 0.163), although this reduction was not sustained by the end of the study (**B**). No significant changes were observed in the scores of the PFS-16 (**C**), GDS (**D**), BDIS (**E**), or PDSS-2 (**F**) after 2 or 6 months of FMT therapy.” GDS-SF, Geriatric Depression Scale Short Forms; PDQ-39, Parkinson’s Disease Questionnaire; PD NMS, Parkinson’s Disease Non-Motor Symptoms Scale; PFS-16, Parkinson’s Disease Fatigue Scale; PDDS-2 Parkinson’s Disease Sleep Scale.

There was a statistically significant reduction in PDQ-39 scores between baseline and 2 months (34.7, SD 15.8 vs 25.3, SD 13.2; *p* = 0.032) and a trend towards a reduction in NMSQ scores (9.5, SD 4.3 vs 7.7, SD 3.4; *p* = 0.163), Fig. 3. However, these improvements were not maintained at 6 months. No statistically significant changes were observed in PFS-16, PDSS-2, BDIS, GDS, or PDSS-2 scores after 2 or 6 months of treatment.

### Efficacy of FMT on Bowel Function

There was no improvement in bowel-related symptoms, as assessed by the 7-day bowel diary, after 2 or 6 months of FMT therapy. However, fewer subjects met the Rome IV criteria for functional constipation at 6 months post-treatment (baseline 9/12 [75%] vs 6 months 7/11 [64%]).

### Dietary optimisation and adequacy during FMT Therapy

Before receiving FMT, subjects’ habitual diets were inadequate in dietary fibre and resistant starch, with a higher total protein intake. After individualized dietary advice, there was a trend toward increased dietary fiber at 8 weeks (mean intake 28.2 g/d ± 9.98 g/d), but this was not sustained at 24 weeks (24.7 g/d ± 9.55 g/d). Three subjects (27%) declined to modify their diet, and three (27%) chose to follow a gluten-free diet.

### Microbiome Changes with FMT Therapy

There was no difference in alpha diversity (Shannon index), richness, or evenness (Pilou index) of the gut microbiome from baseline in patients treated with FMT for 2 and 6 months. While minor changes were observed in some individual patients, most reverted to baseline by month 6, and aggregated results were non-significant, indicating no overall changes throughout the study.

## Discussion

This is the first study to evaluate the safety and tolerability of a 6-month FMT enema therapy course in patients with mild to moderate PD, showing that FMT is safe and well-tolerated. Apart from one serious adverse event (SAE) unrelated to FMT, adverse events were mild, self-limiting, and did not lead to treatment discontinuation. Most subjects rated FMT as safe and tolerable. These safety findings are consistent with several recent studies in which patients with mild to moderate PD received FMT through various routes—including oral capsules, colonic delivery, or nasointestinal administration—as either a single dose or multiple doses over up to three months, with follow-up periods extending to 12 months^11–14^. Across these studies, side effects were predominantly mild, gastrointestinal in nature, and self-limiting. Similar side effect profile has been well established in FMT studies for alternative indications, mainly for the management of Clostridium Difficile Infection^15^. In particular, a systematic review encompassing 5688 FMT procedures across 129 studies for various indications, showed diarrhoea and abdominal cramping to be the most common adverse effects with the majority mild and transient in nature^15^.

Although this study was not primarily designed to assess efficacy, a significant improvement in QOL scores was observed at 2 months following FMT. Additionally, trends toward improvement were noted in several non-motor scales, including the non-motor symptom questionnaire (NMSQ), UPDRS Parts I & II, and PDSS-2 scores, although these effects were not maintained at 6 months. In a recent placebo-controlled trial, participants who received FMT twice weekly for 12 weeks similarly reported greater subjective improvement in symptoms such as constipation, falls, sleep disturbances, and fatigue, as measured by a 100-point visual analogue scale^11^. Nevertheless, other studies have not demonstrated statistically significant improvements in standardized non-motor symptom measures among FMT-treated participants compared to placebo^12,14^. These inconsistencies across studies underscore the need for more rigorous, well-powered trials to determine the existence and extent of non-motor therapeutic benefits of FMT.

The therapeutic effects of FMT on motor function in PD remain inconclusive. Recent placebo-controlled trials investigating the potential motor benefits of FMT in PD have yielded mixed results. Notably, substantial methodological heterogeneity across studies—such as differences in FMT administration route, treatment frequency and duration, and follow-up periods—may account for the variability in outcomes^11–14^. In the GUT-PARFECT trial, patients receiving a single nasojejunal FMT demonstrated a significant improvement in UPDRS motor scores at 12 months compared to placebo^12^. Similarly, DuPont et al. reported transient, objective motor improvements on physical examination in patients treated with twice-weekly oral FMT over a three-month period^11^. However, a recent systematic review and meta-analysis of three randomized controlled trials, encompassing a total of 145 patients, found no significant effect of FMT on motor symptoms compared to placebo^16^.

In the present study, the greatest improvement in non-motor symptoms was observed at 2-months, corresponding with the induction phase of therapy involving weekly or fortnightly FMT enemas. This finding suggests that more frequent dosing may be necessary to sustain therapeutic benefit. However, the optimal frequency and duration of FMT required to achieve meaningful motor and non-motor improvements in PD remains to be established. In a study by DuPont et al., patients receiving twice-weekly oral FMT for 12 weeks reported subjective improvements in symptoms commonly associated with PD, although these were not supported by sustained objective motor improvements on physical examination^11^. Conversely, Bruggeman et al. demonstrated that a single nasojejunal FMT resulted in a significant improvement in UPDRS motor scores at 12 months compared to placebo, with the most pronounced difference occurring between the 6- and 12-month follow-up periods^12^. In contrast, Scheperjans et al. found no significant motor benefit following a single colonoscopically administered FMT over a similar 12-month follow-up period^14^. The divergent outcomes of these studies may be attributed to methodological differences—particularly in the route of FMT delivery (nasojejunal vs oral vs colonoscopic)—underscoring the need for future research to determine the optimal mode, frequency, and duration of FMT administration in PD.

Although no significant improvement in bowel symptoms was observed in the present study, as assessed by 7-day bowel diary, a reduction in the proportion of subjects meeting the Rome IV criteria for functional constipation was noted at 6 month follow-up (75% at baseline vs. 64% at 6 months). Recent studies have reported improvements in constipation following FMT, including changes in bowel transit time and motility index, potentially attributable to increased gut microbiome diversity^11,12^. However, our study did not demonstrate significant changes in alpha diversity, richness, or evenness of the gut microbiome, which we hypothesise may be due to the enema-based delivery method employed. It has been suggested that colonic FMT is less likely to alter gut microbiota composition in the small intestine compared to nasojejunal FMT. Furthermore, since the vagus nerve only partially innervates the colon (approximately two-thirds), the jejunum, being fully innervated, may serve as a more optimal site for FMT delivery^12^. We did not control for dietary intake throughout the treatment duration which likely impacted on the success of FMT engraftment, with factors such as high-fibre intake and low processed sugar diets associated with greater success^17^.

We acknowledge that the open label nature of our study lends it to risk of observer and observer bias, overestimation of therapeutic effects as well as potential placebo effect, all of which has the potential to hinder the interpretation of results. We recognise the inherent risk of a type 1 error in the absence of a blinded placebo-controlled arm to the study. In addition, the study’s small sample size limited its ability to detect significant effects.

Our study is the first to utilize the Hauser Motor Diary to evaluate the potential motor benefits of FMT in PD. The Hauser Motor Diary is a reliable and valid tool in PD clinical research and may offer superior sensitivity and accuracy in detecting changes in dopaminergic ON/OFF time compared to the UPDRS. We observed a transient improvement in OFF time following FMT therapy, warranting further investigation in larger, placebo-controlled trials, including changes in Hauser Motor Diary as a primary outcome. The single-centre, single-investigator design of this study minimized inter-site and inter-investigator variability in data collection. Furthermore, the six-month duration of therapy in this study is among the longest in existing trials, demonstrating that prolonged FMT therapy in PD is safe and laying the groundwork for future studies exploring treatment durations beyond 6–12 months. Additionally, further research is required to determine the most effective mode of FMT administration (i.e. colonic vs nasojejunal vs oral), with careful consideration of the balance between tolerability and efficacy.

## Methods

### Study Participants

Twelve participants aged over 30 with a history of PD were recruited for this pilot study between June 2021 and November 2022. Key inclusion criteria were: (i) diagnosis of idiopathic PD for ≤10 years, as per the UK Parkinson’s Disease Society Brain Bank criteria; (ii) a positive dopaminergic response, defined by a ≥ 33% reduction in Movement Disorders Society-Unified Parkinson’s Disease Rating Scale (MDS-UPDRS) III motor score between OFF and ON-dopaminergic medication states; and (iii) motor fluctuations with at least 2 h of daily OFF time on at least two consecutive days. Subjects with mild cognitive impairment (Montreal Cognitive Assessment score <26) were excluded. Participants were required to maintain stable PD therapy for 30 days before and throughout the study.

### Study Design

This was a prospective open-label, single centre study conducted by the Neurology and Gastroenterology Units of the Central Adelaide Local Health Network, Adelaide, Australia. Subjects underwent clinical assessment at baseline (pre-FMT), and at 2- and 6-months post FMT treatment commencement.

### FMT Treatment Regimen

FMT enemas were sourced from Biomebank, a Therapeutic Goods Administration (TGA)-accredited provider based in South Australia (ARTG #399066). The FMT donor screening, collection, and manufacturing followed the standards outlined in Therapeutic Goods Order No. 105 – Standards for FMT^18^. The FMT was administered by a clinical nurse as a 50 mL enema containing 12.5 g of donor faeces. Subjects were instructed to retain the FMT for at least 30 min in the right lateral position. The intervention consisted of an induction phase with 6 donor FMT enemas over 8 weeks (weeks 0, 1, 2, 3, 5, 7), followed by a maintenance phase with 4 monthly donor FMT enemas over 4 months (months 3, 4, 5, 6), totalling 6 months of FMT therapy.

### Study Assessments

Daily OFF time was measured using Hauser motor diaries, completed by subjects for 48 consecutive hours. Subjects rated their motor state every half hour as “on without dyskinesia,” “on with non-troublesome dyskinesia,” “on with troublesome dyskinesia,” “off,” or “asleep.” Daytime ratings were contemporaneous, and overnight scores were made retrospectively.

Subjects were assessed using the Movement Disorder Society Unified Parkinson’s Disease Rating Scale (MDS-UPDRS) and the modified Hoehn and Yahr Scale. The MDS-UPDRS includes 4 domains: Part I (non-motor aspects of daily living), Part II (motor aspects of daily living), Part III (motor examination), and Part IV (motor complications).

Subjects attended baseline visits in the “OFF medication” state after overnight withdrawal of dopaminergic medication. The MDS-UPDRS Part III score was assessed in both “OFF” and “ON” medication states, approximately 1 h after the usual morning dose of dopaminergic medication. For the 2- and 6-month visits, no pre-visit motor state stipulation was required. Motor examinations were performed by a single movement disorders specialist.

Safety and tolerability were monitored through adverse event reporting, patient experience questionnaires, and visual analogue scales. Subjects completed an adverse event questionnaire at each visit. Subjects completed a 2-point Likert scale questionnaire at each visit rating FMT’s acceptability, tolerability, and safety based on preconceived opinions and during treatment. Six non-motor questionnaires were completed, including the Geriatric Depression Scale Short Form (GDS-SF), Parkinson’s Disease Questionnaire (PDQ-39), Parkinson’s Disease Non-Motor Symptoms Scale (PD NMS), Parkinson’s Disease Fatigue Scale (PFS-16), Beck Depression Inventory, and Parkinson’s Disease Sleep Scale (PDSS-2). Across the study, subjects also reported subjective changes in motor symptoms, falls, constipation, and cognitive function. Bowel function was assessed using a 7-day diary, and microbiome analysis was performed at all timepoints. Constipation was defined by the Rome IV criteria for Bowel Disorders.

Subjects received dietary education from an academic dietitian at -14 days pre-FMT and at 2- and 6-month visits. They completed a 3-day weighed food diary, and data were analysed for energy, macronutrients, fibre, and micronutrients.

A Safety Review Committee (SRC) comprising experts in gastroenterology (RB) and neurology (TK, MD) was established to ensure participant safety.

### Microbiome Analysis

Stool samples were collected in OMNIGene gut tubes (DNA Genotek) and sent for shotgun metagenomic sequencing at a depth of 40 M 150 bp paired-end reads on a DNBseq NanoBall platform.

### Statistical Methods

Data are presented as mean ( ± standard deviation) unless otherwise noted. Comparisons were analysed using a mixed-effects model to account for missing data, with Tukey’s multiple comparisons tests to compare timepoints. A p-value of <0.05 was considered statistically significant. Dietary data were analysed using one-way ANOVA for normally distributed data. Statistical analyses were performed with GraphPad Prism version 10.0.2. A convenience sample size was used for this pilot study.

Microbial makeup was profiled using standard bioinformatics tools (MetaPhlan V4), and microbiome analysis was conducted at BiomeBank, Australia to assess species richness, evenness (Pilou index), and alpha diversity (Shannon index). Microbiome indices were analyzed using a mixed-effects model with Tukey’s tests. Missing stool samples and questionnaires were handled with appropriate statistical methods.

## Supplementary information

CONSORT-2010-Checklist

## Data Availability

The data that support the findings of this study are available from the corresponding author upon reasonable request.
